# Supplementary material for: EnzML: multi-label prediction of enzyme classes using InterPro signatures
Source: BMC Bioinformatics. 2012 Apr 25;13:61. doi: 10.1186/1471-2105-13-61 (PMC3483700; doi:10.1186/1471-2105-13-61)
Supplement: Addtional file 5 — The Java code to format the data files, evaluate and predict. The file enzml_java_code.tar.gz contains the Java code used to format database data to ARFF and XML formats, to execute cross and train-test (jackknife) evaluations and to record evaluation results to database. More information is included in the readme.txt file and the Javadoc files. The code can be used with a MySQL database. To use a different database software, other JDBC drivers might be required. [file 1471-2105-13-61-S5.gz › java_code/enzml2011/doc/uk/ac/ed/inf/taweka/mulan/package-summary.html]

uk.ac.ed.inf.taweka.mulan


---


|  |  |  |
| --- | --- | --- |
| **Overview** \| **Package** \| Class \| **Use** \| **Tree** \| **Deprecated** \| **Index** \| **Help** | |  |
| **PREV PACKAGE**   **NEXT PACKAGE** | **FRAMES**    **NO FRAMES**     **All Classes** |  |


---

## Package uk.ac.ed.inf.taweka.mulan

| **Class Summary** | |
| --- | --- |
| **AttributeUtils** |  |
| **InstanceUtils** | Class |
| **MulanArff** | Generates an Attribute Relation Format File (ARFF) in Mulan format (multiple class labels per instance). |
| **MulanArffProperties** | Class |
| **MulanAttributeFactory** | Class |
| **MulanDataSetDbLoader** | Loads data from database to a Mulan data factory |
| **MulanDataSetGenerator** | Class |
| **MulanDataSetManager** | This class generates a data set (instances, attributes, classes) in Mulan style (for multi-label machine learning). |
| **MulanInstancesFiller** | Class |

---


|  |  |  |
| --- | --- | --- |
| **Overview** \| **Package** \| Class \| **Use** \| **Tree** \| **Deprecated** \| **Index** \| **Help** | |  |
| **PREV PACKAGE**   **NEXT PACKAGE** | **FRAMES**    **NO FRAMES**     **All Classes** |  |


---
